# Abdominal Aortic Aneurysm and Liver Fibrosis: Clinical Evidence and Molecular Pathomechanisms

**DOI:** 10.3390/ijms26073440

**Published:** 2025-04-07

**Authors:** Mohamad Jamalinia, Amedeo Lonardo, Ralf Weiskirchen

**Affiliations:** 1 Gastroenterohepatology Research Center, Shiraz University of Medical Sciences, Shiraz 41100, Iran; mohamadjamalinia@gmail.com; 2 Department of Internal Medicine, Azienda Ospedaliero-Universitaria of Modena, 41126 Modena, Italy; 3 Institute of Molecular Pathobiochemistry, Experimental Gene Therapy and Clinical Chemistry (IFMPEGKC), RWTH University Hospital Aachen, D-52074 Aachen, Germany; rweiskirchen@ukaachen.de

**Keywords:** aortic aneurysms, epidemiology, liver fibrosis, MASLD, molecular pathogenesis, risk factors

## Abstract

To stimulate further research, this review summarizes studies linking liver fibrosis with the risk of abdominal aortic aneurysms (AAA). AAA is defined as a permanently weakened and dilated abdominal aorta, which develops due to inflammation of the tunica media, activation of the renin–angiotensin–aldosterone system, immune system activation, and coagulation disorders. Typically asymptomatic, AAA is often incidentally detected through imaging done for abdominal symptoms or as part of screening programs. AAA follows a variable course and has a mortality rate strongly dependent on age and sex. Risk factors for AAA include age, male sex, ethnicity, family history of AAA, lifestyle habits, arterial hypertension, dyslipidemia, and comorbid atherosclerotic cardiovascular disease. Conversely, individuals with type 2 diabetes, female sex, and certain ethnicities are at a reduced risk of AAA. Liver fibrosis, resulting from chronic liver diseases owing to varying etiologies, is increasingly recognized as a potential contributor to AAA development. Evidence increasingly indicates that metabolic dysfunction-associated steatotic liver disease (MASLD) and other chronic liver conditions may intensify inflammatory pathways shared with AAA, thereby potentially exacerbating AAA progression. This review specifically examines the epidemiology and risk factors associated with the link between AAA and liver fibrosis. It also highlights potential pathomechanisms, including systemic inflammation, oxidative stress, and extracellular matrix remodeling, which may contribute to both conditions. Although these findings underscore significant overlaps in risk profiles, additional research is needed to clarify whether type 2 diabetes, female sex, and certain ethnicities truly confer protection against AAA or if this association is influenced by other confounding variables. Ultimately, addressing these open questions will help guide targeted therapeutic interventions and the identification of novel biomarkers to predict disease progression.

## 1. Introduction

An abdominal aortic aneurysm (AAA) is defined as a weakened and permanently dilated abdominal aorta, commonly infrarenal, with a diameter ≥30 mm [1]. The natural course of AAA varies, and mortality due to AAA is strongly age- and sex-dependent [1]. Typically asymptomatic, AAA is rarely detected through physical examination and is usually identified through imaging techniques performed for abdominal symptoms or during screening programs. These screenings are crucial, as elective (usually endovascular) repair significantly reduces morbidity and mortality compared to emergency interventions for ruptured aneurysms [1,2]. Several risk factors contribute to the development of AAA, including age, male sex, ethnicity, family history of AAA, lifestyle habits (such as tobacco smoking), arterial hypertension, dyslipidemia, and comorbidities such as coronary or peripheral artery disease. Conversely, individuals with type 2 diabetes mellitus (T2DM) and certain ethnicities (such as African American and Asian) are at a reduced risk of AAA [3]. In affluent countries, the prevalence and mortality due to AAA are decreasing, while both are increasing in low-income countries [4]. Although the exact cause of AAA remains unknown, factors that trigger an inflammatory state at the level of the tunica media, along with the renin–angiotensin–aldosterone system (RAAS), immune system activation, and coagulation disorders, are all believed to play a causal role [5,6].

Liver fibrosis occurs due to the excessive buildup of extracellular matrix proteins in response to chronic liver injury caused by various hepatotoxic agents [7]. It acts as a precursor to advanced forms of chronic liver disease (CLD), including cirrhosis, portal hypertension, liver failure, and hepatocellular carcinoma [7]. Beyond the liver, fibrosis is increasingly being recognized as an indicator of overall health, especially in assessing the risk of cardiovascular disease [8]. Evidence suggests that cardiovascular health is significantly influenced by sex, reproductive status, and, notably, the function of the liver within the liver-kidney-metabolic axis [9]. The cardiovascular–kidney–metabolic (CKM) syndrome concept, introduced by the American Heart Association, identifies steatotic liver disease (SLD) as an early risk factor for multisystem organ damage, including cardiovascular disease [10]. Metabolic dysfunction-associated SLD (MASLD) is closely linked to an increased risk of fatal and nonfatal cardiovascular events, congestive heart failure, and the development of T2DM. This explains why cardiovascular mortality is a leading cause of death among non-cirrhotic MASLD individuals, highlighting the significant pathogenic role of metabolic dysregulation in this patient population [11]. However, it is still uncertain whether AAA and atherosclerotic cardiovascular disease share common pathomechanisms or are distinct conditions [12]. Some experts suggest that these two pathologies may be more different than previously believed [13]. Given that recent research has focused on the role of liver fibrosis as a risk factor for the development and progression of AAA [14], this review critically examines published studies linking liver fibrosis and health with the risk of developing and progressing AAA. The aim is to promote further investigation in this field. To find relevant articles, we searched the PubMed/PMC database using keywords such as “cirrhosis”; “liver fibrosis”; “aneurysms”; and “aorta”. Additionally, we found useful cross-references by reading selected articles and utilizing additional articles from our personal database. Marfan syndrome (MS) is beyond the scope of this article, as most aortic aneurysms in this disease are dissecting aneurysms in the thoracic aorta, while AAA is relatively rare among these patients [15].

## 2. Epidemiology and Risk Factors

Critical analysis of the published studies on the association of AAA among individuals with liver fibrosis, as summarized in Table 1 [14,16,17,18,19,20], highlights several key epidemiological insights and methodological challenges. These include geographic limitations, study design heterogeneity, and a lack of ethnic diversity, all of which may affect the generalizability of findings.

### 2.1. Methodological Limitations

There appears to be a significant under-representation of global ethnic heterogeneity, with only a few countries being typified (Italy, Israel, USA, China, and Iran) and studies missing from other parts of the world (notably Africa, South America, Central, and Northern Europe, India, Japan, and Australia). Additionally, the single study from the USA lacks data regarding ethnic diversity [18]. Given the well-established ethnic variability in both AAA and liver fibrosis prevalence, future research should prioritize more diverse cohorts [21,22].

Another notable limitation is the predominance of retrospective study designs, which are inherently prone to selection bias and confounding factors. Additionally, differences in AAA treatment strategies—ranging from open surgical repair to endovascular aneurysm repair (EVAR)—further complicate study comparisons. A recent meta-analysis of 13 studies suggests that while EVAR is associated with lower short-term mortality, open repair may provide better long-term survival and reduced reintervention rates [23]. Therefore, the local availability of these techniques may potentially affect sex differences in the various outcomes of treated AAA.

### 2.2. Liver Fibrosis Due to MASLD

It is currently unclear whether the increasing prevalence of liver fibrosis as a contributing factor to AAA is directly linked to metabolic dysfunction-associated steatotic liver disease (MASLD) or if it simply reflects the decreasing prevalence of other chronic liver diseases, such as viral hepatitis. Between 1990 and 2019, global mortality from hepatitis B and C decreased [24]. During the same time frame, T2DM, hypertension, obesity, hypercholesterolemia, and MASLD have emerged as significant global health concerns [25], explaining why MASLD-related fibrosis and -cirrhosis are the primary cause of CLD in many countries. Additionally, MASLD and AAA share a “common soil” in the CKM syndrome [26]; therefore, further research is necessary to determine if MASLD-induced fibrosis directly impacts AAA risk or if they simply coexist due to shared metabolic dysfunction.

### 2.3. Shared Risk Factors Between AAA and MASLD

**Figure 1:**
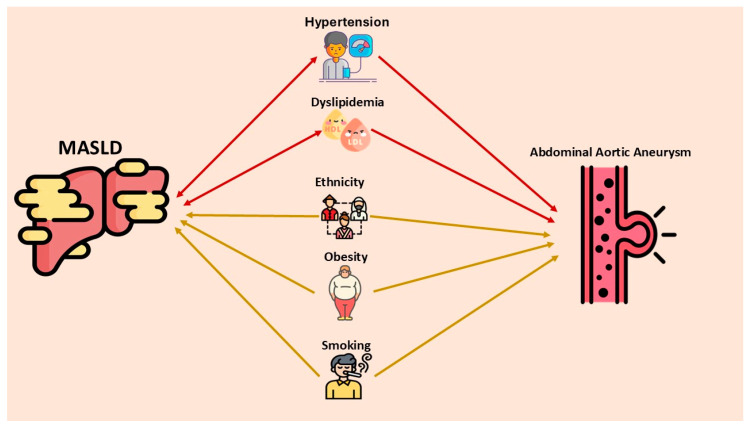
Shared risk factors between MASLD and abdominal aortic aneurysm (AAA) and their complex associations. Arterial hypertension and dyslipidemia are bidirectionally linked to MASLD and also increase the risk of AAA (red double-headed arrow). Ethnicity, smoking, and obesity are unidirectionally associated with both MASLD and AAA (gold single-headed arrow). MASLD—metabolic dysfunction-associated steatotic liver disease.

Several macro-mechanisms appear to link MASLD and AAA, including shared cardiometabolic risk factors, systemic inflammation, endothelial dysfunction, and extracellular matrix remodeling. A study in Denmark involving 11,294 individuals (56% women) who underwent computed tomography angiography of the aorta revealed that subclinical aortic aneurysms (AA) (both thoracic and abdominal) were four times more common in men than in women [27]. In both genders, advancing age and body surface area (a better indicator of metabolic mass than body weight) [28] are risk factors for AAAs in any location [27]. Additionally, AAAs were strongly associated with hypercholesterolemia and smoking (OR = 2.4 (95% CI: 1.6–3.6) and 3.2 (95% CI: 1.9–5.4), respectively) [27]. Figure 1 provides an overview of the potential shard risk factors and their role in the link between MASLD and AAA.

#### 2.3.1. Hypercholesterolemia

An experimental study in LDL receptor-deficient (LDr -/-) mice found that hypercholesterolemia accelerates both the initiation and progression of AAAs in AngII-infused genetically engineered male mice [29]. In agreement, a robust line of research has established that cholesterol crystals accumulating within hepatocyte lipid droplets are strongly associated with metabolic dysfunction-associated steatohepatitis (MASH) in humans, promoting disease progression through lipotoxic and pro-inflammatory molecular pathways [30,31,32].

In humans, epidemiological studies on hypercholesterolemia have yielded conflicting results. For example, in their case-control study of 98 cases with AAA compared to 102 AAA-free controls, Blanchard et al. [33] found that neither clinical hypercholesterolemia nor serum levels of total cholesterol, low-density lipoprotein cholesterol, and high-density lipoprotein cholesterol were associated with AAA. Conversely, the prospective Tromsø study conducted in a cohort of 2035 men and 2310 women followed for 7 years found hypercholesterolemia to be significantly associated with an increased incidence of AAA [34]. Therefore, this area requires further investigation.

#### 2.3.2. Smoking

Tobacco smoking is more prevalent among men, contributing to sex differences in AAA risk [35,36]. A systematic review and meta-analysis of 23 prospective studies confirmed a strong association between smoking and AAA development [37]. It is worth noting that smoking is also a recognized risk factor for the development and the fibrotic progression of MASLD. The pathogenic effects of smoking in MASLD are mediated through multiple mechanisms, including altered lipid metabolism, disrupted hepatic AMPK/SREBP/SIRT1/mTOR signaling in both hepatic and extrahepatic tissues, and hepatocellular injury. Additionally, smoking promotes oxidative stress, inflammatory pathways, apoptosis, and hepatic fibrogenesis through various mechanisms [38]. Whether additional sex- and/or gender-related factors beyond tobacco exposure influence these associations remains an open question, warranting further investigation.

#### 2.3.3. Obesity

A modeling meta-analysis of 54 articles across 19 studies has documented the notion that obesity is one of the risk factors for AAA (meta-OR 95% CI 1.20 (1.18–1.23) I^2^ 68.2 P for Q test 0.043 [39]. Visceral fat promotes vascular inflammation through pro-inflammatory cytokines (IL-6, TNF-α, and MCP-1) [40], while elevated leptin levels contribute to smooth muscle cell apoptosis and aortic wall weakening [41]. Obesity-related insulin resistance and oxidative stress further drive endothelial dysfunction and arterial stiffness, exacerbating AAA risk [42]. Additionally, dysregulated matrix metalloproteinases (MMP-2, MMP-9) facilitate extracellular matrix degradation, leading to aortic wall remodeling and aneurysm formation [43].

#### 2.3.4. Diabetes

A paradoxical protective effect of diabetes on the development and progression of abdominal aortic aneurysms (AAA) has been known for years. Larsen et al. [44] conducted a meta-analysis of 23 population-based screening studies and found that the overall adjusted OR for AAA among those with T2DM was 0.63 (CI 0.59–0.69) before 2000 and 0.69 (CI 0.57–0.84) after 1999. However, the mechanisms underlying this inverse association remain poorly understood. Potential explanations include elevated glycated hemoglobin levels and the use of specific antidiabetic medications, such as metformin, which have been associated with reduced AAA growth [44,45,46]. These effects may be mediated through biological pathways influencing extracellular matrix volume and glycation, advanced glycation end-product synthesis, inflammation, oxidative stress, and intraluminal thrombus biology [47,48]. At this time, a spurious protective effect of T2DM on AAA cannot be ruled out. This would be explained by T2DM carrying an increased mortality of patients owing to concurrent complications before they develop AAA.

#### 2.3.5. Arterial Hypertension

A meta-analysis of 21 cohort studies involving over 5.4 million participants found that arterial hypertension (HTN) increases the risk of AAA by 66% (RR: 1.66, 95% CI: 1.49–1.85 [49]. Chronic HTN induces vascular remodeling, endothelial dysfunction, and arterial stiffness, weakening the aortic wall and promoting aneurysm formation [50]. Beyond its role in AAA pathogenesis, HTN is also a key driver of liver fibrosis in MASLD. Sustained elevation in blood pressure exacerbates hepatic microvascular dysfunction, leading to hypoxia, oxidative stress, and inflammation—key contributors to hepatic fibrogenesis [51]. Longitudinal studies show that high-normal BP (aHR: 1.75, 95% CI: 1.06−2.90) and hypertension (aHR: 2.02, 95% CI: 1.27−3.21) independently predict liver fibrosis progression [52]. The RAAS also plays a crucial role in this link, as its overactivation in HTN fosters fibrogenic signaling via TGF-β and MMP activity, contributing to both aneurysm expansion and hepatic fibrosis [53]. Additionally, endothelial dysfunction and arterial stiffness reduce hepatic perfusion, further promoting fibrotic progression [54].

#### 2.3.6. Aortic Distensibility Is Altered and Endothelial Dysfunction Occurs in MASLD

Growing evidence suggests that MASLD contributes to vascular dysfunction by altering aortic elasticity and impairing endothelial function. Isilak et al. [55] were the first to report that MASLD patients exhibit abnormal aortic elastic properties due to multiple hemodynamic abnormalities associated with insulin resistance. In the same year, Vlachopoulos et al. [56] showed that MASLD is associated with arterial stiffness and endothelial dysfunction. Of concern, postmenopausal women with MASLD display particularly pronounced arterial stiffness, independent of the metabolic syndrome [57], highlighting the role of sex-related factors in vascular impairment. Additionally, the NAFLD-fibrosis score (NFS) correlates with early markers of vascular damage [58], reinforcing the link between hepatic fibrosis and systemic vascular dysfunction.

Further studies have elucidated the mechanisms underlying endothelial dysfunction in MASLD. In 2017, Persico et al. conducted a pioneering proof-of-concept study [57] that showed that endothelial nitric oxide synthase (eNOS) dysfunction occurs selectively in platelets rather than hepatic tissue. This was later confirmed by Al-Hamoudi et al. [59], who found that MASH subjects exhibit reduced flow-mediated dilation (FMD) of the brachial artery, indicating systemic endothelial impairment. Moreover, elevated GGT and ALT serum levels have been proposed as predictive biomarkers for endothelial dysfunction [60], further supporting the role of GGT as a biomarker of cardiometabolic risk [61].

Emerging evidence also suggests a connection between liver dysfunction and aortic dilation. Calanchini et al. [62] found a novel association between abnormal liver function tests (LFTs) and aortic dilation in women with Turner syndrome, a population at increased risk of fibrosing MASLD [63]. These findings highlight the broader implications of MASLD beyond hepatic pathology, underscoring its potential role in the progression of vascular diseases, including aortic aneurysms.

## 3. Molecular Pathomechanisms

AAA and liver fibrosis may appear as distinct clinical entities, each primarily affecting a different organ system. However, growing evidence suggests that these conditions share several key pathogenic features, including chronic inflammation, oxidative stress, and aberrant extracellular matrix remodeling, all of which accelerate tissue damage and disease progression. This section describes the molecular pathomechanisms of AAA and how these processes intersect with fibrogenic pathways in the liver, offering insights into potential common therapeutic targets and highlighting the need for interdisciplinary research on these complex and often interconnected disorders. The precise pathomechanism of AAA is not fully understood yet, but it is clear that AAA is a condition that is triggered by inflammation, oxidative stress, different soluble mediators, genetic factors, and more. The contribution of these factors will be discussed in the following.

### 3.1. Pathomechanisms of Abdominal Aortic Aneurysm

AAA and liver fibrosis are both significant health conditions that pose considerable risks to patients worldwide. Understanding their underlying mechanisms is crucial for developing effective prevention and treatment strategies. In this chapter, we discuss the pathophysiology of AAA and its relation to liver fibrosis, highlighting key processes such as inflammation, oxidative stress, matrix remodeling, and cellular signaling pathways that contribute to the progression of these diseases.

**Figure 2:**
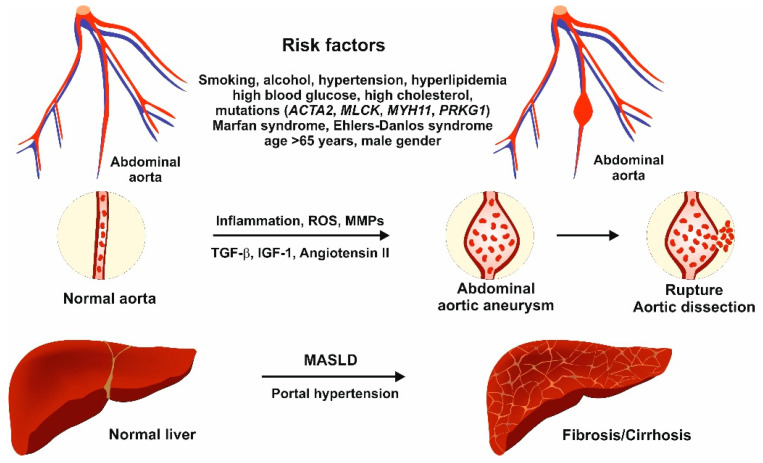
Pathomechanisms of abdominal aortic aneurysm (AAA). Numerous risk factors are proposed for the development of AAA. Inflammation, reactive oxygen species (ROS), the altered composition of matrix metalloproteinases (MMPs), transforming growth factor-β signaling, IGF-1 signaling, and angiotensin II signaling all contribute to the transition from a normal aorta to AAA. Aortic dissection occurs when the innermost layer of the aorta ruptures, allowing blood to flow between the layers of the aortic wall. Additionally, there is a significant relationship between MASLD and AAA, as MASLD has a broad impact on the cardiovascular system.

The pathogenesis of AAA involves several interrelated mechanisms (Figure 2). Chronic inflammation plays a pivotal role; inflammatory cells such as macrophages, T lymphocytes, and many other immune cells infiltrate the aneurysmal wall and contribute significantly to local tissue damage [64,65]. These immune cells secrete pro-inflammatory mediators like tumor necrosis factor-alpha (TNF-α), interleukin-6 (IL-6), interleukin-1β (IL-1β), interleukin-23 (IL-23), and MMPs, which further amplify inflammation and stimulate matrix degradation through various pathways [65].

Oxidative stress induced by reactive oxygen species (ROS) production is another critical factor in AAA development [66]. ROS can activate MMPs and induce apoptosis in vascular smooth muscle cells (VSMCs), weakening the aortic wall and increasing susceptibility to aneurysm formation [66]. This oxidative stress is often exacerbated by risk factors such as smoking and hypertension, which further enhance ROS generation, leading to endothelial dysfunction and AAA progression.

MMPs are enzymes responsible for extracellular matrix (ECM) remodeling. In patients with AAA, elevated levels of MMPs lead to degradation of collagen and elastin fibers within the arterial wall, critical structural components necessary for maintaining vascular integrity. The imbalance between MMP activity and their natural inhibitors, known as tissue inhibitors of metalloproteinases (TIMPs), results in excessive ECM breakdown, leading to progressive vessel dilation, which ultimately can lead to AAA. In particular, the upregulation of MMP-2 and MMP-9 has been identified as a key event occurring during aneurysmal growth [67].

### 3.2. Gene Mutations in the Pathogenesis of Abdominal Aortic Aneurysm

Genetic predispositions play a crucial role in determining susceptibility to developing AAAs through mutations affecting ECM components, vascular remodeling, and inflammatory responses. Variants in genes associated with these processes have been implicated. Mutations in specific genes significantly increase the risk of AAA and dissections by affecting three main pathways: the BMP/TGF-β pathway [68,69,70], ECM integrity [71], and smooth muscle contractile function [72]. The TGF-β pathway is vital for smooth muscle cell (SMC) and vascular biology, but its precise mechanisms remain unclear. While mutations in this signaling pathway are typically loss-of-function mutations associated with AAA, paradoxically, elevated TGF-β signaling has been observed in end-stage aneurysmal tissue from affected patients [73,74].

Initially, increased TGF-β was thought to drive AAA pathogenesis, based on mouse models of Marfan syndrome (MFS), where fibrillin 1 (FBN1) mutations led to enhanced activation of latent TGF-β [75]. While TGF-β neutralization showed some effectiveness in these models [76], blocking this pathway resulted in severe complications such as aortic dissection or rupture, indicating that TGF-β signaling is essential for maintaining aortic integrity early in life [77]. FBN1 plays a crucial role not only in regulating TGF-β signaling but also as a key structural component of microfibrils that support elastin and SMCs within the aorta. Mutations in the FBN1 gene reduce fibrillin-1 production and increase its breakdown.

Furthermore, genes involved in SMC contractile function, such as α-actin (ACTA2) and myosin heavy chain 11 (MYH11), encode proteins essential for forming contractile filaments [78]. Mutations in these genes can impair SMC contraction and relaxation, affecting vascular integrity by impairing key regulatory proteins, such as myosin light chain kinase (MLCK) and protein kinase G type 1 (PRKG1). Consequently, disruptions in these pathways weaken the aortic wall’s structural integrity, increasing susceptibility to dissection or rupture associated with aneurysms [79].

Most identified mutations in the *ACTA2* gene are DNA mutations that impair actin polymerization or ATP hydrolysis, which are essential for SMC contraction. These mutations include missense and premature truncating variants [80]. Aortic tissues of patients with ACTA2 mutations exhibit characteristics such as proteoglycan accumulation, disorganized SMCs, and fragmented elastic fibers [81]. Patients with ACTA2 mutations frequently experience higher rates. These mutations are also linked to an increased risk of occlusive vascular diseases, including strokes and coronary artery disease [82]. In particular, R179H mutation has been associated with multisystemic smooth muscle dysfunction, broadly affecting vascular wall stress adaptation and contributing to aneurysm formation [83].

Studies using mouse models indicate that simply deleting the *ACTA2* gene does not directly cause aneurysms. Instead, aneurysms develop only when exposed to angiotensin II, highlighting the interaction between genetic predisposition and environmental factors in aneurysm development [84]. Notably, ACTA2 mutations can lead to aortic dissection even without prior aneurysm formation [80].

Myosin light chain kinase (MLCK), encoded by the MYLK gene, has been implicated in the aortic phenotype [85]. MLCK is responsible for phosphorylating regulatory light chains (RLC), which enhances myosin II ATPase activity, endocytosis, and the formation of stress fibers [86]. This phosphorylation is crucial for SMC contraction and helps regulate arterial pressure. Consequently, mutations in the MYLK gene or artificial downregulation of MLCK have been shown to significantly reduce MLCK activity [87,88]. While most blood vessels can tolerate a defined level of reduction, the aorta, subjected to high forces, is particularly vulnerable and may exhibit pathological changes.

The myosin heavy chain 11 gene (*MYH11*) encodes smooth muscle myosin heavy chain (SM-MHC), an essential contractile protein produced by SMCs. Mutations in MYH11, like those in ACTA2, disrupt SMC contraction, which is vital for maintaining the stability of the aortic wall [89]. Specific mutations located in the C-terminal coiled-coil region of SM-MHC have been associated with AAA, likely through a dominant negative mechanism [86]. The resulting phenotype includes increased pulse wave velocity and decreased aortic compliance, indicating reduced elasticity due to SMC dysfunction, ultimately leading to dissection. Histological examinations of affected patients show focal fibromuscular dysplasia in the vasa vasorum, like inflammatory patterns seen in other thoracic aortopathies [90].

Additionally, alterations in insulin-like growth factor 1 (IGF-1) and angiotensin II signaling pathways indicate mechanisms distinct from other TGF-β-related mutations. IGF-1 signaling promotes contractile protein production and SMC proliferation, which may contribute to the higher incidence of occlusive vascular diseases seen in these patients [91]. Notably, one study found that MYH11 mutations did not segregate with thoracic aortic aneurysm and dissection (TAAD) within a Dutch family, implying that unidentified genetic modifiers may influence aneurysm development.

The *PRKG1* gene encodes the type I cGMP-dependent protein kinase (PKG-1), which plays a crucial role in regulating SMC contraction by modulating phosphatases that dephosphorylate the RLC [92]. The predominant isoform of PKG-1 in SMCs is activated by elevated levels of cGMP. A gain-of-function mutation at the cGMP binding site (Arg177Gln) alters PKG-1’s structure, rendering its inhibitory domain inactive and leading to continuous activation, which decreases RLC phosphorylation and results in SMC relaxation [93]. Additionally, a case report identified another mutation in the ATP binding domain associated with aortic dissection [94]. However, clinical information regarding PRKG1 mutations is limited but indicates a severe aortic phenotype.

Interestingly, genome-wide association studies (GWAS) identified or confirmed several AAA-associated genetic variants, many of which overlap with atherosclerosis risk factors [95]. These loci have previously been associated with either low-density lipoprotein cholesterol such as the low-density lipoprotein receptor (*LDLR*), proprotein convertase, subtilisin/Kexin type 9 (*PCSK9*), sortilin (*SORT1*), lipoprotein a (*LPA*), apolipoprotein E (*APOE*), or triglycerides including apolipoprotein A-V (*APOA5*), or tribbles pseudokinase 1 (*TRIB1*) [95]. Moreover, a study investigating 31 familial AAA patients and 12 sporadic AAA patients identified forty-seven variants in the genes encoding collagen, type III α1 (*COL3A1*), EGF-containing fibulin-like extracellular matrix protein 2 (*EFEMP2*), fibrillin 1 (FBN1), *MYH11*, myosin light chain kinase (*MYLK*), transforming growth factor-β2 (*TGFB2*), transforming growth factor-β receptor type I (*TGFBR1*), and transforming growth factor-β receptor type II (*TGFBR2*), suggesting that additional genes are involved in the pathogenesis of AAA [96].

### 3.3. Relation Between Abdominal Aortic Aneurysm and Liver Disease

The pathogenesis of liver fibrosis involves interconnected mechanisms, with the activation of hepatic stellate cells (HSCs) playing a central role. Under normal conditions, HSCs remain quiescent, storing vitamin A [97]. However, upon exposure to injury signals from damaged hepatocytes, they become activated and transform into myofibroblast-like cells capable of producing excess collagen along with other ECM components. This leads to progressive fibrogenesis, resulting in structural remodeling and impaired hepatic function [97].

Oxidative stress plays significant roles in both contexts. In the liver, it induces hepatocyte apoptosis while activating HSCs via signaling pathways involving transforming growth factor-β (TGF-β) [98]. Chronic oxidative stress creates a pro-fibrotic environment, fostering progression toward cirrhosis if not addressed promptly. ROS are drivers of fatty liver disease [99]. Similarly, patients with aortic dissection have altered lipid metabolism, characterized by elevated levels of total cholesterol and low-density lipoprotein (LDL) [100]. Oxidized LDL (OxLDL) can lead to apoptosis and necrosis of endothelial cells (ECs), as well as the expression of adhesion molecules and the release of chemokines [101]. These factors contribute to inflammatory cell infiltration in the aortic intima, accompanied by increased ROS production and decreased endothelial nitric oxide synthase (eNOS) expression, resulting in endothelial dysfunction [102]. This shift may play a critical role in atherogenesis and subsequent aneurysm formation and rupture.

Conversely, high-density lipoprotein (HDL) appears to have a protective effect against aortic dissection [103]. HDL promotes cholesterol efflux, maintains aortic wall integrity, and reduces inflammatory cell-EC interactions by inhibiting the expression of VCAM-1 and ICAM-1 [104]. Additionally, HDL regulates eNOS activity, increasing nitric oxide production through pathways mediated by endothelial SR-BI receptors and S1P-related MAPK and PI3K/Akt signaling [105]. Reduced HDL levels eliminate this protective mechanism, potentially increasing aneurysm risk.

Chronic inflammation drives progression toward advanced stages where ongoing recruitment and activation of immune cells perpetuate cycles, causing further damage and promoting HSC activation, contributing to the overall fibrotic response [97]. Similar mechanisms observed during AAA development can also be seen here, highlighting how persistent inflammation acts as a unifying feature linking these two disparate conditions together. Therefore, targeting inflammatory pathways might offer therapeutic potential across both entities, paving the way toward improved management strategies.

As discussed, both AAAs and liver fibrosis share several common mechanisms: dysregulation of ECM synthesis versus degradation determines outcomes directly related to fibrotic processes. An imbalance between ECM deposition and degradation remains a critical determinant driving the advancement of the respective diseases’ severity, ultimately affecting patient outcomes adversely if left unchecked, necessitating close monitoring and interventions aimed at restoring balance whenever possible.

In conclusion, understanding the intricate interplay among various contributors, from inflammation and oxidative stress to molecular signaling pathways, provides valuable insights into the mechanisms governing the pathogenesis behind AAA and hepatic fibrosis. By elucidating these shared mechanisms, further research may pave new avenues for targeted therapies aimed at mitigating serious health conditions associated with each entity, ultimately improving patient outcomes.

## 4. Conclusions

This narrative review has examined the principal epidemiological features and molecular pathways potentially linking AAA and liver fibrosis. Several unresolved questions remain, including the seemingly protective effect of female sex, certain ethnicities, and diabetes on AAA risk. Moreover, similarities and differences between atherosclerotic cardiovascular disease and AAA require further investigation, as do the shared and distinct mechanisms underlying AAA and thoracic aortic aneurysms, particularly in relation to liver fibrosis owing to MASLD and non-MASLD CLD.

Findings discussed in the present manuscript may suggest, but they do not definitely demonstrate, a cause-and-effect association between fibrosing MASLD and AAA. Indeed, an underlying set of shared systemic risk factors, by activating common molecular/cellular mechanisms in different organs or tissues, can promote either pathology independently. Although it is tempting to propose a common origin for fibrosing MASLD and AAA (or at least an interaction between the two), fibrosing MASLD and AAA should be considered comorbidities to consider in the prediction of clinical outcomes and proposal of management strategies. One concrete methodology to ascertain or refute a cause-and-effect relationship could be Mendelian randomization analysis (MRA). By using genetic variants with a known biological function to explore the effects of modifiable exposure on an outcome, MRA promises to overcome the limitations of observational studies and provide stronger evidence for causal inference because gene variants are independent of many factors that may confound observational associations.

Future studies should also focus on investigating specific therapeutic interventions that target these common pathways while also exploring novel biomarkers that can predict disease progression. This will enable clinicians to better stratify patients based on their individualized risk profiles, enhancing the precision medicine approaches in current practice settings. Ensuring that optimal care is delivered effectively and efficiently will benefit populations comprehensively, addressing the needs that arise from the complexities inherent in today’s modern healthcare landscape. A better understanding of the complex cross-talks occurring among key players, such as inflammation and oxidative stress, promises to provide valuable insight into those molecular signaling pathways determining the shared pathways underlying both AAA and hepatic fibrosis. Ensuring interdisciplinary collaboration between vascular surgeons, hepatologists, cardiologists, and researchers in molecular medicine can accelerate the development of targeted diagnostic tools and treatments to lower AAA-associated morbidity and mortality while simultaneously addressing liver-related comorbidities. Assessment of these mechanisms may pave the way for a broader range of innovative therapies and prophylactic strategies aimed at mitigating the burden of these serious and interrelated health conditions.

## Figures and Tables

**Table 1:** Overview of published studies documenting the association of liver fibrosis with abdominal aortic aneurysm [14,16,17,18,19,20].

Author Year [Ref.]	Origin	Methods	Findings	Conclusions
Marrocco-Trischitta J, 2011 [16]	Italy	Between January 2001 and March 2006, 1189 consecutive patients underwent elective open repair of infrarenal AAA, with 24 (2%) of them having cirrhosis. The patients with cirrhosis included 23 males and 1 female with a mean age of 68 ± 7 years. They were retrospectively stratified based on the CTP and MELD scores. Operative variables, perioperative complications, and survival rates were compared to those of 48 matched non-cirrhotic controls, and the impact of CTP and MELD scores on midterm survival was assessed in cirrhotic patients using the Kaplan–Meier log-rank method.	Major perioperative complications occurred to a similar extent among cirrhotic patients and controls. However, the duration of surgery, requirements of intraoperative blood transfusion and duration of hospital stay were all higher in patients with cirrhosis (*p* = 0.007; *p* = 0.040; *p* < 0.0001, respectively). CTP class B cirrhotic subjects had higher requirements for intraoperative blood transfusions (*p* = 0.029). 2-year actuarial survival rates were 77.4% among patients with cirrhosis vs. 97.8% in controls (log-rank test, *p* = 0.026). CTP class B and a MELD score ≥10 were associated with reduced mid-term survival rates (*p* < 0.0001 and *p* = 0.021, respectively).	Although elective AAA open repair in patients with relatively compensated cirrhosis was safely performed, the reduced life expectancy of patients with cirrhosis and a MELD score ≥10 suggest avoiding this procedure in this specific patient cohort.
Mahamid M, 2019 [17]	Israel	A retrospective case-control study was conducted with 495 AAA patients and 500 age- and sex-matched controls.	The prevalence of FLD was higher among AAA patients than in controls (48.9% vs. 21.2% *p* < 0.005). LRA after adjusting for confounding factors showed that AAA (men: OR 1.29, 95% CI 1.17, 1.49, *p* = 0.001; women: OR 1.23, 95% CI 1.06, 1.43, *p* = 0.002), obesity (men: OR 1.32, 95% CI 1.17, 1.59, *p* < 0.001; women: OR 1.32, 95% CI 1.07, 1.52, *p* = 0.012), hypertension (men: OR 1.23, 95% CI 1.13, 1.46, *p* = 0.001; women: OR 1.13, 95% CI 1.00, 1.33, *p* = 0.045), MS (men: OR 1.31, 95% CI 1.19, 1.53, *p* = 0.001; women: OR 1.28, 95% CI 1.16, 1.42, *p* = 0.002) were associated with NAFLD/NASH. The prevalence of liver cirrhosis was 1.23%; and subjects with obesity, diabetes, hypertension, and AAA had an increased risk of cirrhosis (OR 1.89, 95% CI 1.18, 3.22, *p* = 0.014; OR 1.27, 95% CI 1.09, 2.72, *p* = 0.0027; OR 2.08, 95% CI 1.29, 3.42, *p* = 0.004; OR 1.73, 95% CI 1.08, 2.87, *p* = 0.027, respectively).	Patients with AAA are at an increased risk of NAFLD/NASH, which may progress to cirrhosis.
Zettervall SL, 2020 [18]	USA	The National Surgical Quality Improvement Program evaluated all nonemergent EVARs from 2005 to 2016. An APRI >0.5 was used to identify significant liver fibrosis, and demographics, comorbidities, and 30-day outcomes were compared between patients with and without fibrosis. Further analysis was performed to evaluate the effect of increasing MELD scores on 30-day outcomes, using MVRA to adjust for baseline differences.	EVAR was performed in 18,484 patients (2286 with liver fibrosis and 16,198 without). Patients with liver fibrosis had an increased 30-day mortality (1.5% vs. 2.4%; *p* < 0.01) and significantly higher rates of major morbidities. At MVA, patients with liver fibrosis had a significant increase in 30-day mortality (OR, 1.5; 95%, CI, 1.1–2.1), re-operation (OR, 1.5; 95% CI, 1.2–1.8), pulmonary complications (OR, 1.6; 95% CI, 1.2–2.0), transfusion (OR, 1.7; 95% CI, 1.5–2.0), and discharge other than home (OR, 1.5; 95% CI, 1.3–1.8). Mortality also increased in parallel with an increase in MELD score (MELD <10, 1.3%; MELD 10–15, 2.3%; MELD >15, 4.7%; *p* < 0.01), and so did major complications (MELD <10, 7%; MELD 10–15, 11%; MELD >15, 15%; *p* < 0.01). These increases persisted in adjusted analysis.	Liver fibrosis is significantly associated with increased mortality and major morbidity after EVAR. The APRI and MELD scores can be used for preoperative risk stratification. Current 30-day morbidity and mortality rates among patients with MELD scores >10 exceed 5%, which is higher than the annual rupture risk for aneurysms <6 cm, supporting the utilization of an increased size threshold of >6 cm before EVAR in patients with liver fibrosis.
Jia Y, 2024 [19]	China	A total of 370,203 participants (36.4% with MAFLD) from the prospective UK Biobank cohort study were followed up for 12.5 years. MAFLD was defined as HS plus metabolic abnormality, T2DM, or overweight/obesity. AAA data was collected using ICD-10 code, and Cox regression was used to analyze the association between MAFLD and AAA.	During follow-up, 1561 (0.4%) of participants developed AAA. In fully adjusted analysis, MAFLD was associated with a significantly higher likelihood of AAA (HR 1.521, 95% CI 1.351–1.712, *p* < 0.001), and the AAA risk increased with the severity of MAFLD fibrosis scores irrespective of sex, weight, alcohol consumption, and PRS. However, these associations were weaker in the elderly or diabetics (*p* for interaction <0.05). MAFLD was not associated with TAA or aortic dissection.	MAFLD was significantly associated with AAA, which has important clinical implications.
Jamalinia M, 2025 [20]	Iran	In a retrospective longitudinal study of 141 consecutive AAA open repair surgery patients (92% male, mean age of 70 years (SD: 11.5)) from October 2016 to September 2021, with a median follow-up of 35 months (IQR: 0.7–56.6), the primary outcome being all-cause mortality, aHRs were calculated for each Fib-4 cut-off between 1.5 and 3.25.	FIB-4 ≥ 2.67 increased mortality by 78% (aHR:1.78, 95% CI: 1.06–3.00). Furthermore, FIB-4 ≥ 2.67 was significantly associated with a baseline aneurysm size ≥ 8cm (aOR: 2.67, 95% CI: 1.17–6.09). FIB-4 was independently associated with higher mortality risk and higher aneurysm size.	Non-invasive assessment of liver fibrosis with FIB-4 may aid in more precise risk stratification and management strategies for AAA patients in clinical practice.
Wang X, 2025 [14]	China	A retrospective analysis of 151 AAA subjects (mean age 69.1 ± 10.5 years) and age- and sex-matched healthy subjects who underwent abdominal CTA and non-enhanced CT scanning from January 2015 to January 2023. AAA subjects were categorized into progression (growth rate > 10 mL/year) and non-progression groups based on growth rate, as well as those with and without NAFLD, based on abdominal CT results. The Kaplan–Meier and Cox regression were used to investigate the association between NAFLD and AAA progression.	A total of 66 out of 151 AAA patients had NAFLD. Over a median of 10.7 months (6.0–76.0 months), 57 patients (37.7%) had AAA progression. The prevalence of NAFLD was significantly higher in the AAA group compared to the control group (43.7% vs. 31.1%, *p* = 0.024). MRA showed NAFLD to be an independent predictor of AAA progression (HR, 4.28; 95% CI, 2.20–8.31; *p* < 0.001). The AUC of combined NAFLD and AAA maximal diameter was 0.857 for predicting AAA progression.	NAFLD assessed with non-enhanced CT independently predicts AAA progression and can help improve the prediction of AAA progression.

List of abbreviations used: AAA—abdominal aortic aneurysm; aHR—adjusted hazard ratios; APRI—aspartate transaminase to platelet ratio index; AUC—area under curve; CI—confidence interval; CT—computed tomography; CTA—computed tomography angiography; CTP—Child–Turcotte–Pugh; EVARs—endovascular aneurysm repairs; FLD—fatty liver disease; HR—hazard ratio; HS—hepatic steatosis; MVA—multivariable regression analysis; MAFLD—metabolic dysfunction-associated fatty liver disease; MELD—model for end-stage liver disease; MVRA—multivariable regression analysis; NAFLD—nonalcoholic fatty liver disease; NASH—nonalcoholic steatohepatitis; PRS—polygenic risk score; T2DM—type 2 diabetes mellitus.

## Data Availability

No new data was generated.

## Abbreviations

The following abbreviations are used in this manuscript:

AAA	abdominal aortic aneurysm
CKM	cardiovascular–kidney–metabolic
CLD	chronic liver disease
ECM	extracellular matrix
EVAR	endovascular aneurysm repair
HSC(s)	hepatic stellate cell(s)
HTN	arterial hypertension
MASH	metabolic dysfunction-associated steatohepatitis
MASLD	metabolic dysfunction-associated steatotic liver disease
MS	Marfan syndrome
MMP(s)	matrix metalloproteinase(s)
RLC	regulatory light chain
RAAS	renin–angiotensin–aldosterone system
ROS	reactive oxygen species
SLD	steatotic liver disease
SMC(s)	smooth muscle cell(s)
T2DM	type 2 diabetes mellitus
TIMP(s)	tissue inhibitor of metalloproteinase(s)
